# Eye movement abnormalities in Parkinson’s disease motor subtypes: a video-oculographic study

**DOI:** 10.1007/s10072-025-08184-w

**Published:** 2025-04-21

**Authors:** Jolanda Buonocore, Alessio Facchin, Marianna Crasà, Giulia Sgrò, Alessia Cristofaro, Aldo Quattrone, Andrea Quattrone

**Affiliations:** 1 Institute of Neurology, Department of Medical and Surgical Sciences, Magna Graecia University, Catanzaro, Italy; 2 Neuroscience Research Center, Magna Graecia University, Viale Europa, Germaneto, 88100 Catanzaro, Italy

**Keywords:** Parkinson’s disease, Motor subtypes, Tremor-dominant, Postural instability and gait difficulty, Video-oculography, Eye movement

## Abstract

**Introduction:**

Eye movement dysfunction has been described in Parkinson’s disease (PD), but differences between tremor-dominant (TD) and postural instability/gait difficulty (PIGD) PD motor subtypes remain poorly understood. The aim of this study was thus to compare video-oculographic (VOG) features between PD motor subtypes.

**Methods:**

Two hundred and four PD patients and 55 age-matched healthy control subjects (HC) were enrolled in this study. PD patients were stratified into PIGD and TD motor subtype groups. VOG amplitude, peak velocity of upward, downward, and vertical saccades, and square wave jerks (SWJ) number and amplitude were compared across groups. Multivariate linear models also investigated associations between VOG parameters and motor severity, gait/balance disturbances, dopaminergic treatment, and cognitive function.

**Results:**

The final cohort included 180 PD patients classified as PIGD (*n* = 121) or TD subtype (*n* = 59) and 55 HC. Both PD subtypes showed reduced upward and downward amplitude compared to HC, with normal peak velocity. PIGD patients exhibited significantly decreased upward saccadic amplitude compared to TD patients, with no differences in other VOG parameters. Moreover, the upward saccadic amplitude was associated with motor severity, particularly slowness of gait and bradykinesia/rigidity scores, as well as with levodopa equivalent daily dose (LEDD) in the PIGD group.

**Discussion:**

This study provides evidence of greater saccadic hypometria in PIGD than in TD patients in upward gaze, contributing to a better understanding of oculomotor impairment in PD. The association of saccadic amplitude with bradykinesia/rigidity severity and LEDD may suggest a role of underlying dopaminergic deficits in ocular dysfunction in PD patients.

## Introduction

Eye movement dysfunction is commonly observed in various movement disorders [1, 2], with specific features in Progressive Supranuclear Palsy (PSP), as highlighted in the most recent MDS clinical diagnostic criteria [3]. Eye movement evaluation is a non-invasive assessment that can be easily performed through simple bedside observation; in addition, video-oculography (VOG) constitutes a highly sensitive, non-invasive method for characterizing ocular movements in neurodegenerative diseases [2, 3]. On these bases, there is a growing interest in identifying the most sensitive and specific VOG parameters to aid in the early detection of oculomotor changes, which may support differential diagnosis or serve as markers of disease progression or prognosis in Parkinsonian syndromes [4–8]. In PSP patients, vertical saccades are typically fragmented, hypometric, and slow, as detected both clinically and by video-oculography; in a previous VOG study, we suggested a VOG index—calculated by multiplying amplitude and peak velocity (AxV) of vertical saccades – as an accurate measure for differentiating PSP from Parkinson’s disease (PD) patients, with higher performances than saccadic amplitude or peak velocity used alone [8]. In PD patients, eye movement clinical examination is often normal. Still, VOG may detect subtle eye movement abnormalities commonly manifesting as increased saccadic latency, mild hypometria, and impaired inhibition of reflexive saccades [9], which may be helpful as progression or prognostic markers [10]. Within the PD population, two primary motor subtypes are recognized: tremor-dominant (TD) and postural instability and gait difficulty (PIGD) [11]. PIGD patients typically show clinical differences in comparison with TD patients, having a more severe disease trajectory, earlier onset of axial symptoms, postural instability, falls, and cognitive decline [11, 12]; however, the impact of different PD motor subtypes on ocular dysfunction in PD remain poorly understood.

In the current study, we enrolled a large cohort of around 200 PD patients to investigate differences in ocular dysfunction across PIGD and TD parkinsonian patients and to explore associations between oculomotor impairment and other clinical features in PD subtypes.

## Methods

### Participants

The study enrolled 204 PD patients and 55 healthy controls (HC). Patients were recruited consecutively between 2021 and 2024 at the Neurology Institute and Neuroscience Research Centre of the University of Catanzaro, Italy. PD diagnosis was made by movement disorder specialists according to the MDS international diagnostic criteria [13]. The 55 HC participants, all over 50 years old, were recruited among the partners of patients. All study participants underwent a neurological examination and a video-oculographic assessment, with PD patients in the “OFF” state (off medications overnight). Clinical data were collected, including the levodopa equivalent daily dose (LEDD), the MDS-Unified Parkinson’s Disease Rating Scale (MDS-UPDRS) scale [14] and the Mini-Mental State Examination (MMSE) [15]. Additionally, all patients underwent a 3 T brain MRI scan with a recently described protocol [16] to rule out secondary causes of Parkinsonism. Exclusion criteria for patients were clinical features suggestive of other diseases and MRI abnormalities such as neoplasms, lacunar infarctions in the basal ganglia, or diffuse subcortical vascular lesions. None of the controls had a history of neurological, psychiatric, or other major medical conditions and were not currently taking any neurological or psychiatric medication.

PD patients were stratified into PIGD and TD motor subtypes according to previously described criteria [17]. In brief, the ratio of the mean MDS-UPDRS tremor score (comprising 11 items) to the mean MDS-UPDRS PIGD score (comprising five items) was used. A ratio of ≥ 1.15 defined TD patients, while PIGD patients had a ratio of ≤ 0.90. Patients with a ratio between 0.90 and 1.15 were classified as indeterminate (*n* = 24) and not included in the analyses. Patients with a positive score in the numerator and a score of zero in the denominator were categorized as TD. Conversely, those with a zero in the numerator and a positive score in the denominator were classified as PIGD [17]. Based on this methodology, we identified 121 patients with PIGD and 59 with TD. The Institutional Review Board of Magna Graecia University, Catanzaro, Italy, approved all study procedures and ethical considerations. All participants provided written informed consent.

### Video-oculographic data acquisition and analysis

As previously described [8, 18], the VOG examination was conducted using the Eye Link portable duo eye tracking system (SR Research Ltd, Mississauga, Ontario, Canada). Participants were comfortably seated with their heads stabilized by an adjustable chin and forehead rest to avoid head movements. Following the manufacturer’s instructions, they faced a computer monitor positioned 50–60 cm away in a dimly lit, quiet, dedicated room. Before data acquisition, a standard 5-point calibration procedure was performed, with calibration targets presented randomly at five different positions on the screen. Then, upward and downward saccades were elicited by the jump of a target (red dot) on the white screen from the central position to a different destination at ± 20° up or downward, and subjects were instructed to track the target jumps as accurately and quick as possible. We employed a previously described reliable acquisition protocol including four recordings: two for upward saccades and two for downward saccades, and the stimulus was presented five times (5 trials) in a row for each recording [8]. A trained physician reviewed all recordings visually, and trials affected by eye blinking were excluded. The ten trials for each direction were averaged to calculate the mean saccadic amplitude and peak velocity values to enhance the robustness of the VOG data. Then, the amplitude and peak velocity of upward and downward were averaged to calculate the VOG parameters of vertical saccades. AxV values were calculated for upward, downward, and vertical saccades as amplitude multiplied by peak velocity [8]. Cut-offs for abnormal values for VOG parameters were defined as 2.5 standard deviations below the mean of the HC group.

A 5-s fixation task was also available for 69 study participants, including 22 PIGD, 14 TD patients, and 33 healthy controls. Participants were instructed to maintain their gaze on a red dot of 1° size displayed at the center of the screen to evaluate the presence of Square-Wave Jerks (SWJ) interrupting fixation. The SWJs were identified as two consecutive saccades in opposite directions, separated by a short time interval and displaying similar magnitude [18]. The visualization of SWJs was performed by tracing a scatterplot image of the horizontal eye position in which the X axis represents the time (0–5000 ms) and the Y axis represents the eye position (± 6° from the fixation point). A semiquantitative assessment of SWJ number and amplitude was performed as previously described in detail [18].

### Statistical analysis

Statistical analyses were conducted using R statistical software (version 4.3.2, R Foundation for Statistical Computing, 2023). Differences in gender distribution were assessed using Fisher’s exact test. Age at examination was compared across the three groups using an ANOVA test followed by pairwise Wilcoxon rank-sum tests. Disease duration was compared between PD subtypes using the Wilcoxon rank-sum test. Differences in clinical scales, dopaminergic treatment, and all video-oculographic parameters were analyzed using ANCOVA, adjusting for sex, age, and disease duration as covariates. P values were corrected for multiple comparisons (Bonferroni). All statistical tests were two-sided, with a significance threshold set at *p* < 0.05. Linear regression analyses were performed to assess the association between VOG oculomotor features and clinical variables (LEDD, MDS-UPDRS-III score, tremor score, gait score, postural instability score, bradykinesia/rigidity score, and MMSE) adjusting for age and sex. Standardized beta coefficients were calculated, and p values for the variable of interest in each model were extracted and corrected for multiple comparisons using the false discovery rate (FDR). Pairwise Cohen D effect size values were also calculated for SWJ comparison across groups due to the lower number of patients included in this analysis, which might affect statistical significance; effect size values were considered negligible (< 0.2), small (0.2–0.5), medium (0.5–0.8) or large (> 0.8).

## Results

### Demographic and clinical data

The final cohort included 180 PD patients with PIGD (*n* = 121) or TD (*n* = 59) motor subtype and 55 age-matched HC. No differences were found in age and sex across groups. PIGD patients showed slightly longer disease duration than TD patients. Overall, disease severity and cognitive status were similar between the two PD subgroups, with higher scores of gait impairment, freezing, postural instability, and bradykinesia/rigidity in PIGD patients and higher tremor scores in TD patients. In line with these clinical differences, PIGD patients also had higher LEDD than TD patients, likely due to the more pronounced bradykinesia and rigidity observed in PIGD patients (Table 1).

**Table 1:** Demographics and clinical data of study participants

Data	PIGD (*n* = 121)	TD (*n* = 59)	HC (*n* = 55)	*P* value
Sex (M/F)	73/48	39/20	24/31	**0.04**^**a**^
Age at examination, years	68.7 (7.7)	66.4 (7.7)	68.4 (7.7)	0.16^b^
Disease duration, years	6.0 (6.9)	4.0 (3.4)	/	** < 0.001**^**c**^
MDS-UPDRS-III score	26 (4–67)	22 (3–66)	/	0.11^d^
PIGD score	0.8 (0.6)	0.3 (0.2)	/	** < 0.001**^**d**^
Tremor score	0.2 (0.2)	0.8 (0.4)	/	** < 0.001**^**d**^
TD/PIGD score ratio	0.3 (0.3)	2.2 (1.0)	/	** < 0.001**^**d**^
Gait score	2.4 (1.2)	1.4 (0.9)	/	** < 0.001**^**d**^
FOG score	0.7 (1.5)	0.1 (0.3)	/	** < 0.05**^**d**^
PI score	1.0 (1.1)	0.3 (0.6)	/	** < 0.001**^**d**^
Bradykinesia-Rigidity score	18.3 (11.4)	12.2 (8.7)	/	** < 0.001**^**d**^
LEDD	475.1 (402.3)	239.2 (277.5)	/	** < 0.001**^**d**^
MMSE	23.5 (3.9)	23.7 (5.2)	/	0.76^d^

Abbreviations: *PIGD*, postural instability and gait difficulties; *TD*, tremor-dominant; *HC*, healthy control subjects; *MDS-UPDRS-III*, Movement Disorder Society—Unified Parkinson’s Disease Rating Scale-part III (Motor Examination); *H&Y*, Hoehn and Yahr; *FOG*, freezing of gait.; *PI*, postural instability; *LEDD*, levodopa equivalent daily dose; *MMSE*, mini mental state examination.

Data are expressed as mean (standard deviation). All tests were two tailed, and the α level was set at *p* < 0.05. Significant *p* values are highlighted in bold.

The tremor score was calculated as the mean of MDS-UPDRS items 2.10, 3.15, 3.16, 3.17 and 3.18, while the PIGD score was calculated as the mean of items 2.12, 2.13, 3.10, 3.11 and 3.12. The TD/PIGD ratio was calculated by dividing the tremor score by the PIGD score: a ratio ≥ 1.15 indicates TD, ≤ 0.90 indicates PIGD, and between 0.90 and 1.15 was considered indeterminate. The gait score included items 2.12 and 3.10, while the FOG score included items 2.13 and 3.11. The PI score was the item 3.12. The bradykinesia-rigidity score included items 3.3, 3.4, 3.5, 3.6, 3.7, 3.8 and 3.14 of MDS-UPDRS-III.

^a^Fisher’s exact test. No significant differences were found in post-hoc analysis.

^b^Analysis of variance (ANOVA)

^c^Wilcoxon rank sum test.

^d^ANCOVA with sex, age and disease duration as covariates.

### Video-oculographic data

The amplitude of upward, downward, and overall vertical saccades was significantly reduced in both PD subgroups compared to HC. Conversely, no differences were found in saccadic peak velocity in any direction between PD patients and HC. In direct comparisons between PIGD and TD patients, the PIGD group exhibited significantly reduced upward and vertical saccadic amplitudes compared to the TD group after adjusting for age, sex, and disease duration. No significant differences were observed between the two PD subgroups in downward saccades. These results suggest more pronounced saccadic hypometria in PIGD patients, particularly in upward gaze (Table 2).

**Table 2:** Video-oculographic features of the study participants

Data	PIGD (*n* = 121)	TD (*n* = 59)	HC (*n* = 55)	*P* value	Post-hoc
Amplitude upward gaze	12.4 (2.5)	13.7 (2.3)	14.8 (2.0)	** < 0.001** ^**a**^	PIGD < HC TD < HC PIGD < TD
Peak velocity upward gaze	336.3 (75.0)	356.1 (79.2)	344.1 (68.5)	0.13^a^	-
AxV upward gaze	4264.4 (1470.2)	4969.2 (1595.1)	5128.0 (1338.5)	** < 0.05** ^**a**^	PIGD < HC PIGD < TD
Amplitude downward gaze	15.5 (1.7)	16.1 (2.0)	17.2 (1.5)	** < 0.05** ^**a**^	PIGD < HC TD < HC
Peak velocity downward gaze	392.3 (76.0)	393.5 (75.9)	381.0 (53.6)	0.88^a^	-
AxV downward gaze	6134.7 (1575.5)	6399.8 (1651.4)	6598.8 (1257.6)	0.28^a^	-
Amplitude vertical gaze	14.0 (1.7)	14.9 (1.8)	16.0 (1.5)	** < 0.001** ^**a**^	PIGD < HC TD < HC PIGD < TD
Peak velocity vertical gaze	364.0 (64.2)	374.2 (68.6)	363.0 (53.8)	0.34^a^	-
AxV vertical gaze	5123.7 (1275.4)	5634.8 (1383.9)	5840.0 (1077.7)	** < 0.05** ^**a**^	PIGD < HC
Data	PIGD patients (*n* = 22)	TD patients (*n* = 14)	Control subjects (*n* = 33)	*P* value	Post-hoc
SWJs number	2.3 (1.7)	2.0 (1.8)	1.3 (1.5)	0.47^a^	-
SWJs mean amplitude	0.6 (0.4)	0.6 (0.5)	0.4 (0.4)	0.13^a^	-
Total SWJs amplitude	1.8 (1.7)	1.6 (1.6)	0.9 (1.1)	0.15^a^	-

Abbreviations: *PIGD*, postural instability and gait difficulties; *TD*, tremor-dominant; *HC*, healthy control subjects; *AxV*, amplitude multiplied by peak velocity; *SWJ*, square-wave jerks. The saccadic amplitude was measured in degrees, the saccadic peak velocity was measured in degrees/sec and the AxV was expressed in degrees^2^/sec. The amplitude of SWJs was expressed in degrees. The total SWJs amplitude was calculated as the sum of all SWJs amplitudes.Data are expressed as mean (standard deviation). All tests were two tailed, and the α level was set at *p* < 0.05. Significant *p* values are highlighted in bold.

^a^ANCOVA with age, sex, and disease duration as covariates, followed by post hoc test with Bonferroni correction.

**Fig. 1:**
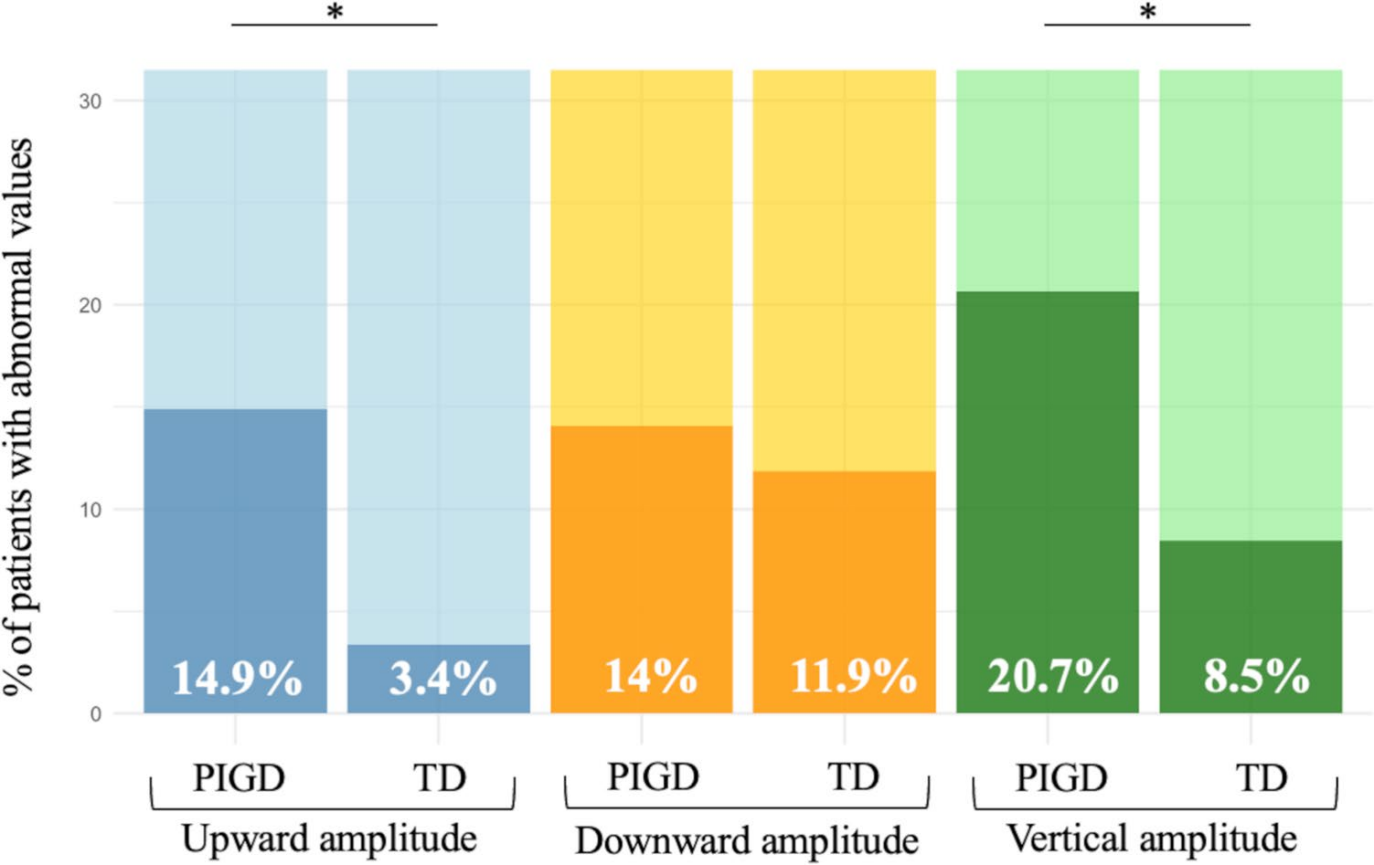
Percentage of patients with abnormal values in upward, downward, and vertical amplitudes in the PIGD (Postural Instability and Gait Difficulty) and TD (Tremor-Dominant) groups. PIGD patients showed significantly higher proportions of abnormal cases in upward amplitude (14.9% vs. 3.4%) and vertical amplitude (20.7% vs. 8.5%) than TD patients. On the other hand, no significant difference was observed in downward amplitude. The asterisk indicates statistical significance with *p* < 0.05

Consistently, a significantly higher percentage of PIGD than TD patients (14.9% and 3.4%, respectively) exhibited a reduction of upward saccadic amplitude beyond normal limits (Fig. 1). Fixation-related parameters did not significantly differ between the PIGD, TD, and HC groups. The effect size was negligible for SWJ number and amplitude between the PIGD and TD groups but medium for PD subgroups versus HC comparisons.

### Associations between oculomotor dysfunction and clinical variables

**Fig. 2:**
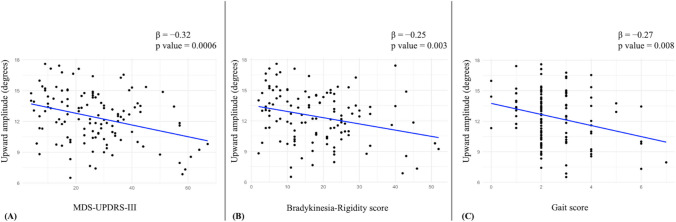
Scatterplots show significant negative associations between upward saccadic amplitude (in degrees) and clinical motor scores in patients with PIGD (Postural Instability and Gait Difficulty). (**A**) Upward amplitude exhibits a significant inverse correlation with the MDS-Unified Parkinson’s Disease Rating Scale motor score (MDS-UPDRS-III). (**B**) A significant negative correlation is observed with the bradykinesia-rigidity score, calculated from items 3.3, 3.4, 3.5, 3.6, 3.7, 3.8, and 3.14 of the MDS-UPDRS-III. (C) Additionally, upward amplitude inversely correlates with the gait score, derived from items 2.12 and 3.10 of the MDS-UPDRS. Blue lines represent regression trends

In the PIGD group, a significant negative association was found between upward saccadic amplitude and overall MDS-UPDRS-III score, suggesting that upward saccadic hypometria was associated with greater motor impairment (Fig. 2). In detail, upward saccadic amplitude was negatively associated with gait (β = − 0.25, *p* = 0.008) and bradykinesia/rigidity scores (β = − 0.27, *p* = 0.003), and with dopaminergic therapy (LEDD) (β = − 0.23, *p* = 0.01), but not with postural instability score, tremor score, and cognitive status in PIGD patients, suggesting that upward saccadic hypometria was associated with the severity of dopaminergic dysfunction in this PD subtype.

## Discussion

In this VOG study, we explored ocular features across PD motor subtypes and identified reduced vertical saccadic amplitude in PIGD patients compared to TD patients, which correlates with the severity of bradykinesia and gait impairment.

Careful clinical examination of eye movements is a valuable complement to neurological and cognitive assessments in Parkinsonian syndromes. Several VOG studies explored ocular impairment in PD patients, and mild saccadic hypometria was the most consistent finding across studies, possibly due to dysfunction of the frontal-basal ganglia-superior colliculus circuit and often observed also in the early stages of the disease [10, 19].

Parkinson’s disease can manifest with a heterogeneous clinical picture, and two opposite motor subtypes can be identified. Patients with a clinical phenotype mainly characterized by gait and balance issues are classified as PIGD (“postural instability and gait difficulty”). In contrast, those with prominent tremor are classified as TD (“tremor-dominant”) [17]. Robust evidence suggested that PIGD is a more malignant PD subtype with more severe dopaminergic degeneration and that PIGD patients show a more severe disease trajectory with greater disability and higher non-motor burden than TD patients [20–22].

In the current study, we demonstrated in a large cohort of PD patients that both PD motor subtypes are characterized by mild hypometria of vertical saccades, involving both upward and downward gaze compared to healthy subjects of similar age. We also provide the first evidence of more severe upward saccadic hypometria in PIGD patients compared to TD subjects after correcting for age, sex, and disease duration. These results are at variance with a recent previous study [19], which failed to detect differences between PIGD and TD in VOG parameters. This discrepancy may be due to different methodology, sample size, or PD disease stage, since this previous study focused on de novo PD patients in the very early stage of the disease, when ocular abnormalities may be subtle and difficult to detect. Unlike saccadic amplitude, we did not find any reduction of saccadic peak velocity in our patients compared to age-matched healthy control subjects. These results align with previous reports in the whole PD population and further demonstrate that velocity reduction in vertical saccades may be a highly specific marker for PSP [7, 8, 23, 24]. Notably, velocity impairment was absent not only in TD patients but also in those with the PIGD subtype, who share several clinical aspects with PSP patients, such as pronounced axial involvement, gait and balance disturbances, postural instability, and falls.

In this study, reduced upward saccadic amplitude, which was the most affected VOG parameter in PD patients and the main differential feature between PIGD and TD subtypes, showed a significant association with motor severity scores. Of relevance, upgaze hypometria was significantly associated with bradykinesia/rigidity scores and the slowness of gait in the PIGD group, and also with the load of dopaminergic therapy (levodopa equivalent daily dose). At the same time, no associations were found with other clinical features, including tremor severity, postural instability, and cognitive status. This result may provide insights into the pathophysiology of hypometria in PD patients. From a pathophysiological perspective, bradykinesia and rigidity have been consistently found associated with the severity of dopaminergic impairment in PD patients, and one study also found an association between saccadic hypometria and putaminal 18 F-FP-CIT uptake in PD [19]; on the other hand, tremor is mainly independent of dopaminergic damage, and postural instability and cognition typically respond poorly to dopaminergic drugs, thus suggesting that they may be caused by deficit of nondopaminergic systems [12, 25, 26]. The main hypothesis on these PD symptoms involves the cholinergic system, specifically the pedunculopontine nucleus for postural instability and the Meynert nucleus for cognition, which are key cholinergic structures projecting to the thalamus, basal ganglia and cortex [26–28]. On these bases, our results corroborate the hypothesis that saccadic hypometria in PD may be due to striatal dopaminergic dysfunction rather than to other neurobiological systems, and the higher saccadic impairment observed in our study well aligns with the more pronounced basal ganglia dopaminergic dysfunction typically observed in PIGD than in TD motor subtypes. These findings also support the potential of saccadic hypometria as a marker of motor dysfunction in PD patients, while other oculomotor deficits, such as increased saccadic latency, may be more associated with concomitant or future cognitive deficits in PD [10, 19].

In the current study, we also systematically assessed square wave jerks (SWJs) during a brief fixation task in a subgroup of 69 individuals. We observed higher frequency and amplitude of SWJs in PD patients than in HC, with around 2 SWJs in PD and 1.3 SWJs within 5 s in control subjects. These results did not reach statistical significance, but this was likely due to the relatively small sample size, as suggested by the medium effect size in both PIGD versus HC and TD versus HC comparisons. On the other hand, very similar values in SWJ parameters were observed across PIGD and TD patients, with no statistical significance and negligible effect size in this comparison. These data demonstrate that PD patients may have slightly more frequent and larger SWJ than HC, with no differences between PD motor subtypes, and suggest different pathophysiological bases for SWJ and saccadic hypometria in PD patients.

This study has several strengths. First, this is one of the largest VOG studies exploring saccadic features in PD patients, involving nearly 200 patients stratified into PIGD and TD subtypes. Second, all patients underwent a standardized clinical assessment and a reliable VOG protocol, which have previously shown reproducibility across multiple assessments [8].

This study has some limitations. First, PD patients did not undergo a post-mortem examination; however, movement disorder specialists made the clinical diagnosis per international diagnostic criteria. Second, VOG assessments were conducted exclusively in the “OFF” medication state to reduce patient variability, precluding evaluation of levodopa’s effects on saccadic parameters. Further research is needed to clarify the role of dopaminergic therapy, given its mixed impact, with some studies indicating improvement in voluntary saccades [29, 30], others reporting no effect on PD-related hypometria [31]. Third, this is a cross-sectional study assessing VOG features in PD patients. Previous evidence suggested that PD subtypes may change over time; thus, longitudinal studies are warranted to better explore saccadic impairment in patients either keeping or switching motor subtype over time, also investigating the possible VOG prognostic value.

In conclusion, this study provides evidence of more severe saccadic hypometria in PIGD than in TD patients in upward gaze and corroborates the hypothesis that saccadic amplitude reduction in PD patients may be related to dopaminergic dysfunction and specifically associated with the severity of motor symptoms, paving the way for a better understanding of oculomotor impairment in different subtypes of PD.

## Data Availability

The data that support the findings of this study are available on request from the corresponding author.
